# Allergen immunotherapy for allergic asthma 

**DOI:** 10.5414/ALX02451E

**Published:** 2024-01-12

**Authors:** J. Christian Virchow, Oliver Pfaar, Marek Lommatzsch

**Affiliations:** 1 Department of Pneumology, Rostock University Medical Centre, University of Rostock, Rostock, and; 2 Clinic for Ear, Nose and Throat Medicine, University Hospital Giessen and Marburg GmbH, Marburg site, Philipps University Marburg, Marburg

**Keywords:** allergen immunotherapy, remission, SCIT, SLIT

## Abstract

Remission is the goal of modern asthma treatment. Allergen immunotherapy (AIT) is an essential component in the armamentarium of personalized asthma therapy. Subcutaneous AIT (SCIT) or sublingual AIT (SLIT) offer the possibility to prevent asthma in patients with allergic rhinitis (reduction of the risk of developing asthma) and the possibility to achieve remission in patients with allergic asthma. Accordingly, AIT should always be considered in patients with asthma and a documented, clinically relevant allergy. However, precise phenotyping of the patient is an essential prerequisite for a success of AIT in asthma.

## Introduction 

Allergen immunotherapy (AIT) is a disease-modifying anti-asthmatic drug (DMAAD) that has the potential to achieve asthma remission without long-term therapy (“off treatment”) [1]. It is therefore also one of the symbols of the current paradigm shift in asthma therapy (away from* symptom control *and towards *symptom prevention *[1, 2, 3, 22]), which is the subject of a separate article in this issue of Allergology. With regard to the history and mechanisms of AIT, reference is made to current reviews [4, 5]. In particular, this article reflects the recommendations of the German AIT guideline from 2022 [6] and the German asthma guideline for respiratory specialists from 2023 [7]. The indication for AIT can be both to prevent the development of asthma and to treat existing allergic asthma. 

## AIT for asthma prevention 

The role of AIT in the treatment of allergic rhinoconjunctivitis is well documented and clinically established [4]. For many years, there was a certain reluctance to use AIT for the prevention and treatment of asthma. Today, AIT is recommended in patients with allergic rhinitis not only for the treatment of allergic rhinitis itself, but also for asthma prevention. Several real-world studies have shown that in patients with allergic rhinitis without concomitant asthma, the prescription of AIT leads to a significant reduction in the risk of asthma development [8, 9]. A long-term study of children with allergic rhinitis (tree and grass pollen allergies) in an unblinded study showed that 2 years after the end of the 3-year treatment phase, the risk of developing asthma was approximately halved [10, 11]. A recent meta-analysis of a total of 17 relevant studies (14,126 patients treated with AIT versus 257,622 untreated controls) showed an average asthma risk reduction of 25% with AIT, with the strongest effects when starting AIT in childhood, in the presence of monosensitization, and with AIT duration over 3 years [12]. In a large placebo-controlled intervention study on the prevention of asthma through AIT with a grass tablet (sublingual immunotherapy (SLIT)) in children and adolescents with allergic rhinitis without concomitant asthma, there was a significant reduction in the number of patients who showed asthma symptoms or had to take asthma medication after completion of AIT (after 3 years) or follow-up (after 5 years) [13], even if the primary endpoint of this study, namely the time until a confirmed asthma diagnosis occurred, was not met (mainly for methodological reasons in the predefined study protocol). For these reasons, it is clinically advisable to offer patients with allergic rhinoconjunctivitis AIT for asthma prevention, especially in the case of mono- or oligosensitizations. In addition to potentially preventing the development of asthma, a desirable side effect of such therapy is a reduction in symptoms and the resulting use of medication for allergic rhinoconjunctivitis. 

### AIT for the treatment of allergic asthma 

**Figure 1:**
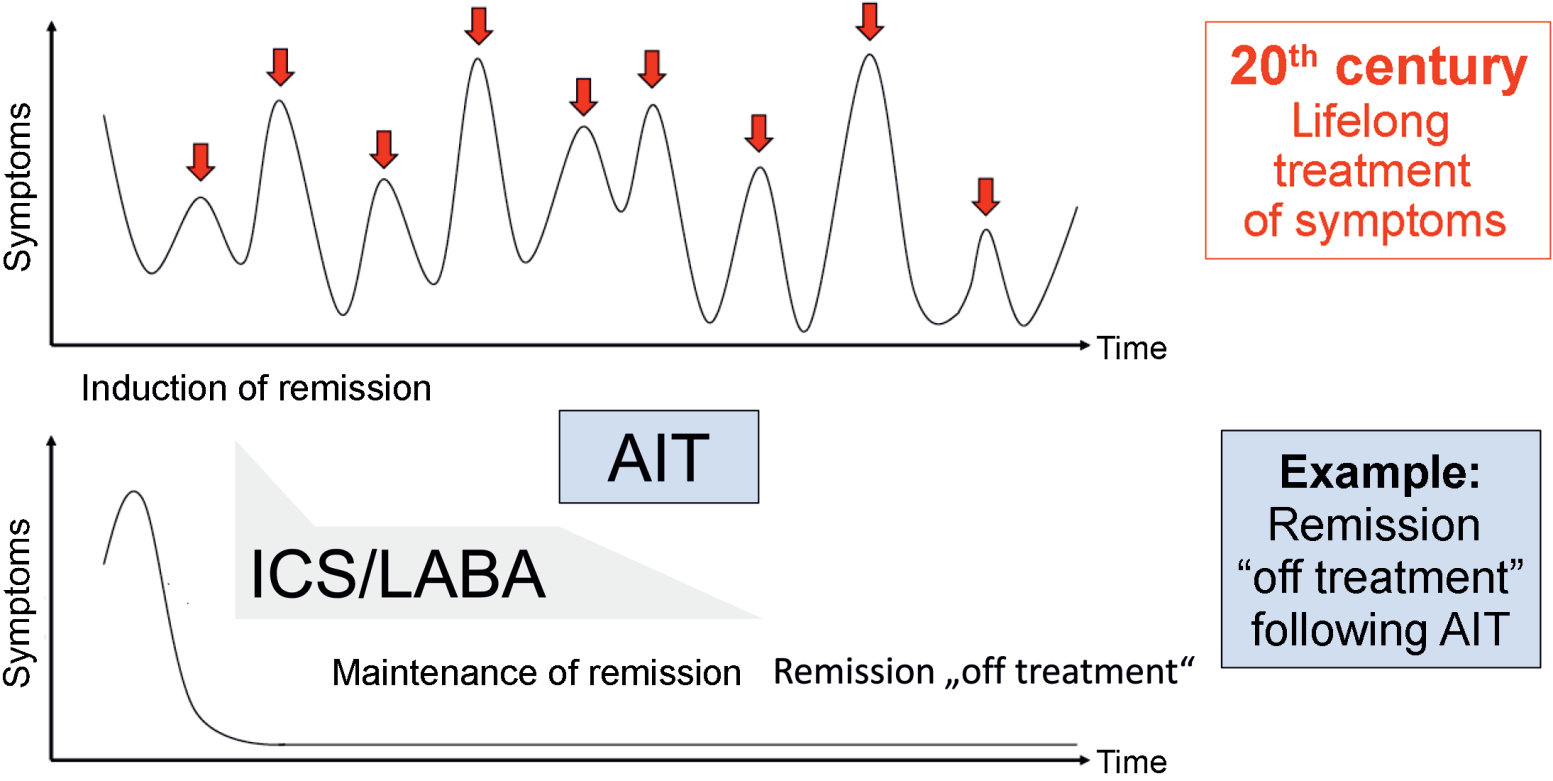
Example of a remission “off treatment” by allergen immunotherapy.

The role of AIT in manifest allergic asthma has been the subject of controversy for many years, partly due to the fact that a highly effective drug therapy with few side effects is available as an alternative for the majority of patients with asthma. On the other hand, AIT is currently the only treatment option for allergic asthma that offers the potential for remission “off treatment”. Recent studies have also shown a significant reduction in exacerbations in patients with severe asthma that is not adequately controlled by medication when the dose of inhaled glucocorticosteroids is reduced [14]. Consequently, AIT in patients with existing allergic asthma is considered a DMAAD that can sustainably improve asthma control, prevent exacerbations, and reduce the need for asthma medication [1]. The ideal goal of AIT is to achieve asthma remission, as mentioned above, in the sense of remission “off treatment” (sustained asthma remission after cessation of AIT) (Figure 1) [1, 6]. 

AIT should not be seen as the primary treatment for asthma, but is carried out after the initiation of adequate drug therapy for asthma. Accordingly, the first step is typically to initiate asthma remission with intensified inhaled therapy (e.g., inhaled corticosteroid (ICS) / long-acting beta agonist (LABA) therapy), particularly as AIT (especially subcutaneous immunotherapy (SCIT)) is contraindicated in uncontrolled asthma for safety reasons. With ongoing inhaled therapy (and improved asthma control), AIT should then be considered as an additional therapy: ideally, inhaled therapy can be reduced during AIT or even stopped completely once AIT has been completed (Figure 1). The indication for AIT in at least partially controlled, IgE-mediated, allergic asthma should therefore always be considered as a treatment option (in addition to allergen avoidance and pharmacotherapy), whereby patients with polyvalent sensitization can also benefit, provided that the allergen being considered for AIT is of proven clinical relevance to the patient’s symptoms. According to German asthma guidelines [7], [15], the use of AIT is in principle possible at all stages: however, the requirements for AIT in asthma (Table 1) [6] must be met. Biologic therapy is not a contraindication for AIT [7]. Pregnancy is considered a contraindication to starting AIT, but AIT can be continued during pregnancy if well tolerated [7]. Overall, clinical experience suggests that AIT should be used primarily in patients with milder forms of asthma, preferably in remission under inhaled therapy, particularly as there is a lack of controlled studies on the success of this therapy in severe asthma. 

### Evidence from clinical studies 

Meta-analyses of various clinical studies indicate a reduction in asthma symptoms and asthma medication consumption following SCIT [16, 17]: however, there is great heterogeneity in the studies with regard to the allergen doses and allergen preparations used, which underlines the importance of a product-specific individual assessment, as recommended in the German guideline [6]. In clinical studies, certain SLIT preparations led to a significant decrease in ICS use [18] and exacerbation rates [14] in patients with asthma with house dust mite allergy (based on these data, GINA recommends a house dust mite SLIT for adult asthma patients with house dust mite allergy in therapy levels 3 and 4 [19]). When using an AIT for the treatment of allergic asthma, the product-specific individual evaluation with corresponding proof of efficacy and safety in clinical studies is therefore of central importance [6]. Accordingly, the AIT guideline from 2022 [6] recommends a product-specific assessment of AIT (tables with the product-specific evidence, approvals and ongoing studies can be found at www.dgaki.de). 

### Evidence from real-world studies 

The large retrospective cohort study REACT analyzed German health insurance data from 2007 to 2017: the analysis showed that AIT prescription in patients with allergic asthma (compared with a control group without AIT prescription) led to a lasting improvement in asthma control, lower medication consumption, and a decrease in the exacerbation rate [20]. In addition, these effects even increased over time after the end of AIT and that there was also an advantage for patients with asthma with regard to the occurrence of pneumonia and hospitalizations. The risk of side effects, in particular the occurrence of anaphylactic shock during the initiation of AIT, was reported to be very low (between 0.03% for SLIT and 0.08% for SCIT). A population-based Danish study compared patients with asthma who received an AIT prescription with patients who did not receive an AIT prescription: in the 3 years following completion of the AIT prescription, there was a sustained reduction in the exacerbation rate (on average by 74% in patients with seasonal allergies and on average by 57% in patients with house dust mite allergies) compared with patients without an AIT prescription [21]. 

### Application route 

SCIT and SLIT are available for the AIT of allergic asthma. The selection of the AIT route is based on several individual criteria, about which patients should be comprehensively informed in the sense of a patient-centered approach (Table 2). With SCIT, most adverse reactions are mild to moderate and can be treated well [6]. Life-threatening systemic reactions are rare if all safety measures are observed. With SLIT, dose-dependent adverse local reactions often occur in the mouth and throat area (especially at the start of therapy) [6]. Anaphylactic systemic reactions are also possible with SLIT, but their occurrence is assumed to be significantly rarer than with SCIT (see also the Anaphylaxis Register of Germany, Austria, and Switzerland: www.anaphylaxie.net). Controlled “head-to-head” comparative studies between SCIT and SLIT are lacking, making it difficult to draw conclusions about their relative effectiveness. Nevertheless, it can be speculated that subcutaneous application may have minor advantages in terms of effectiveness at the cost of a higher side-effect rate, while sublingual application may be slightly less effective with greater safety of use. 

## Conclusion 

AIT in the form of subcutaneous or sublingual application has been an established option for the treatment of allergic rhinoconjunctivitis for decades. There is increasing evidence that AIT both reduces the risk of asthma development and has a disease-modifying effect on existing allergic asthma (in the sense of a DMAAD) and can contribute to achieving asthma remission. AIT therefore represents a classic component of personalized precision medicine for allergic asthma. 

## Funding 

None. 

## Conflict of interest 

J.C. Virchow states that he has given independent, honored lectures for AstraZeneca, Avontec, Bayer, Bencard, Bionorica, Boehringer-Ingelheim, Chiesi, Cipla, German Remedies, Essex/Schering-Plough, Genzyme, GSK, Janssen-Cilag, Leti, Lupin MEDA, Merck, MSD, Mundipharma, Noramed, Novartis, Nycomed/Altana, Pfizer, Providens, Regeneron, Revotar, Sandoz-Hexal, Sanofi-Aventis, Stallergens, TEVA, UCB/Schwarz-Pharma, Zydus/Cadila and independently advised on Advisory Boards Avontec, Boehringer-Ingelheim, Cypla, Chiesi, Essex/Schering-Plough, Genzyme, GSK, Janssen-Cilag, MEDA, MSD, Mundipharma, Novartis, Regeneron, Revotar, Roche, Sanofi-Aventis, Sandoz-Hexal, TEVA, UCB/Schwarz-Pharma as well as research funding from the German Research Foundation, the state of Mecklenburg-Vorpommern, GSK, MSD. 

O. Pfaar acknowledges study grants and/or honoraria and/or travel support from ALK-Abelló, Allergopharma, Stallergenes Greer, HAL Allergy Holding B.V./HAL Allergie GmbH, Bencard Allergie GmbH/Allergy Therapeutics, Lofarma, ASIT Biotech Tools S.A., Laboratorios LETI/LETI Pharma, GlaxoSmithKline, ROXALL Medizin, Novartis, Sanofi-Aventis and Sanofi-Genzyme, Med Update Europe GmbH, streamedup! GmbH, Pohl-Boskamp, Inmunotek S.L., John Wiley and Sons, AS, Paul-Martini-Stiftung (PMS), Regeneron Pharmaceuticals Inc, RG Aerztefortbildung, Institut für Disease Management, Springer GmbH, AstraZeneca, IQVIA Commercial, Ingress Health, Wort&Bild Verlag, Verlag ME, Procter&Gamble, ALTAMIRA, Meinhardt Congress GmbH, Deutsche Forschungsgemeinschaft, Thieme, Deutsche AllergieLiga e.V., AeDA, Alfried-Krupp Hospital, Red Maple Trials Inc, Royal Danish Consulate General, Hannover Medical School, ECM Expro&Conference Management, Dresden University of Technology, Lilly, Paul Ehrlich Institute (PEI), all within the last 36 months. He is a member of the EAACI Excom, member of the extended board of the DGAKI; coordinator, main or co-author of various guidelines and position papers on rhinology, allergology and allergen immunotherapy. 

M. Lommatzsch states that he has received research support from DFG, GSK, Astra Zeneca as well as honoraria for lectures and consultancy work from ALK, Allergopharma, Astra Zeneca, Bencard, Berlin-Chemie, Boehringer-Ingelheim, Bosch, Chiesi, Circassia, GSK, HAL Allergy, Janssen-Cilag, Leti, MSD, Mundipharma, Novartis, Nycomed/Takeda, Sanofi, Stallergenes, TEVA, UCB. 

**Table 1.** Indication for allergen immunotherapy in asthma. Adapted from [17].

Prerequisites for allergen immunotherapy in asthma
Clear causal relationship between respiratory symptoms and corresponding allergen exposure
Evidence of a corresponding allergen-specific sensitization
Use of compounds whose efficacy in patients with asthma has been proven in controlled clinical trials
Contraindication: uncontrolled asthma and/or an FEV_1_ < 70% of the predicted value

**Table 2.** Criteria for selecting the allergen immunotherapy application route. Adapted from [6].

	**PRO**	**CONTRA**
Subcutaneous route	Medical application = certainty of administration	Regular visits to the doctor (time-consuming)
	Frequent doctor/patient contact > regular monitoring of the course of the AIT, side effects and underlying illness(es) of the patients	Possible anxiety-inducing injections
		At least 30 minutes monitoring time after the injection
		Risk of systemic allergic reactions (very rare)
		Risk of local side effects (frequent)
Sublingual Route	Not painful	Risk of local side effects (very common, usually mild and self-limiting)
	Can be administered at home (usually first application at the doctor’s office with 30-minute monitoring)	Usually daily application necessary over a longer period (pre/co-seasonal i.e. several months or perennial > daily “reminder”)
	Small number of visits to the doctor/physician required	Mucosal contact over 2 minutes and patient motivation required (check especially with children)
	Very low risk of systemic reactions (lower than with SCIT)	
